# Gestational Diabetes Mellitus Alters Cytokine Profiles and Macrophage Polarization in Human Placenta

**DOI:** 10.3390/ijms262210867

**Published:** 2025-11-09

**Authors:** Martalice Ribeiro Barbosa, Gabriela Feres de Marchi, Kênia Maria Rezende Silva, Danielle Cristina Honorio França, Marcondes Alves Barbosa da Silva, Jakeline Ribeiro Barbosa, Laura Valdiane Luz Melo, Eduardo Luzía França, Adenilda Cristina Honorio-França

**Affiliations:** 1 Postgraduate Program in Basic and Applied Immunology and Parasitology, Federal University of Mato Grosso, Barra do Garças 78600-000, MT, Brazil; enf.martalice@gmail.com (M.R.B.); keniarezende1@gmail.com (K.M.R.S.); daniellechfranca@gmail.com (D.C.H.F.); 2 Institute of Biological and Health Science, Federal University of Mato Grosso, Barra do Garças 78600-000, MT, Brazil; gabrielamarchi67@gmail.com; 3 Institute of Health Science, Federal University of Rondonópolis, Rondonópolis 78736-900, MT, Brazil; marcondesfarma@hotmail.com (M.A.B.d.S.); lauravaldiane@hotmail.com (L.V.L.M.); 4 Fundação Oswaldo Cruz—Fiocruz, Brasília 70904-970, DF, Brazil; jakelinebarbosa@gmail.com

**Keywords:** gestational diabetes mellitus, placenta, cytokines, type 1 macrophages, type 2 macrophages

## Abstract

Gestational Diabetes Mellitus (GDM) is a metabolic condition characterized by glucose intolerance, which manifests or is diagnosed for the first time during pregnancy. Hyperglycemia associated with GDM can induce a systemic and local inflammatory environment, directly affecting the maternal–fetal interface, particularly the placenta. The placenta, in turn, plays a central role in immune modulation and can alter cytokine and immune cell expression in response to metabolic stress. This study aimed to evaluate levels of inflammatory cytokines and the profiles of type 1 (M1) and type 2 (M2) macrophages in placentas from pregnant women with GDM. Forty placental samples were analyzed and divided into two groups: pregnant women with GDM (n = 20) and normoglycemic pregnant women (n = 20). The villous and extravillous portions were separated and analyzed for cytokine levels by flow cytometry and for macrophage immunophenotyping. The results showed a significant increase in IL-6, IL-8, IL-10, and IL-12P70 levels in the placentas of mothers with GDM, whereas IL-1β and TNF-α were reduced in the extravillous portion of this group. In addition, a higher percentage of CD14+ cells and M2 macrophages was observed, especially in the villous portion of the placentas of pregnant women with GDM. These findings suggest that gestational hyperglycemia modulates the placental immune response, altering cytokine levels and macrophage polarization patterns. GDM influences the placental immunological microenvironment, which can contribute to alterations in placental function and increased risks to fetal development. The data underscore the placenta’s role as an immunoregulatory organ and highlight the need for greater attention to inflammation associated with GDM in maternal and child health.

## 1. Introduction

Gestational diabetes mellitus (GDM) is a pregnancy-specific disease defined by glucose intolerance with onset or first diagnosis during pregnancy. This condition has become increasingly prevalent worldwide and is recognized as a major public health problem due to its strong association with maternal and fetal complications, such as macrosomia, preeclampsia, and long-term risk of type 2 diabetes in both mother and child [1,2,3]. These adverse outcomes may be linked to placental immune dysregulation. The hyperglycemic microenvironment alters cytokine and immune cell profiles in GDM [4,5], thereby impairing trophoblastic invasion, spiral artery remodeling, and nutrient transport, ultimately contributing to placental insufficiency, excessive fetal growth, and the development of preeclampsia [6,7,8,9].

The underlying pathophysiology of GDM is multifactorial, involving chronic low-grade inflammation, oxidative stress, and insulin resistance [10,11,12], which may contribute to immune dysregulation during pregnancy.

The placenta plays a crucial role in maintaining maternal–fetal homeostasis, serving as both an endocrine and immunologically active organ. It is a transient structure with distinct compartments that mediate maternal–fetal exchange and immune regulation. The villous compartment serves as the main interface between the maternal and fetal circulations, facilitating the transfer of nutrients and gases and modulating immune tolerance. The extravillous compartment contains trophoblasts that invade the maternal decidua and remodel spiral arteries, ensuring adequate placental perfusion [13]. GDM induces region-specific structural and immunological changes, influencing trophoblastic invasion, vascular remodeling, and local cytokine balance differently across placental regions [14]. Moreover, hyperglycemia-driven inflammation modifies placental cytokine secretion and cellular activity [4]. Thus, analyzing cytokine profiles separately in villous and extravillous compartments may provide a more precise understanding of the immune responses associated with GDM.

In pregnancies complicated by GDM, this placental environment is significantly altered. Previous studies have demonstrated increased levels of proinflammatory cytokines, dysregulation of trophoblast function, and impaired vascular remodeling [15,16,17]. These immunological and structural changes may compromise maternal–fetal tolerance and contribute to adverse obstetric outcomes, such as fetal growth abnormalities and placental insufficiency.

Placental macrophages, particularly those located in the decidua and villous stroma, play a crucial role as a key component of the local immune microenvironment. These cells exhibit phenotypic plasticity and can transition between classically activated (M1) and alternatively activated (M2) macrophage profiles in response to local stimuli [18,19]. In normal pregnancies, the M2 phenotype predominates, facilitating maternal-fetal tolerance and an anti-inflammatory balance [19]. However, in the context of GDM, increasing evidence suggests a polarization shift toward a proinflammatory M1 profile, contributing to placental dysfunction through cytokine imbalance and tissue remodeling abnormalities [20,21,22,23].

Emerging evidence indicates that macrophage polarization within the placenta is compartment-specific. Distinct immune profiles have been reported between the villous and extravillous regions, suggesting that local immunological regulation may occur in response to the functional demands of each placental zone [24,25,26]. Nevertheless, the effects of GDM on regional macrophage polarization in human placental tissues remain poorly understood. Clarifying this compartment-specific modulation may enhance understanding of the immunopathology of GDM.

This study aimed to evaluate the impact of gestational diabetes mellitus (GDM) on cytokine levels and macrophage polarization in the villous and extravillous compartments of the placenta. The hypothesis was that GDM induces specific immunomodulation in each placental region, characterized by distinct macrophage polarization patterns and cytokine levels in the villous and extravillous compartments. By characterizing the distribution of immune cells and inflammatory mediators in term placentas from diabetic and normoglycemic pregnancies, this study seeks to elucidate the immunopathological mechanisms in different placental compartments associated with GDM.

## 2. Results

### 2.1. Clinical Characteristics of Pregnant Women and Newborns

The clinical characteristics of pregnant women from both groups normoglycemic (ND) and gestational diabetes mellitus (GDM) included in the analyses are presented in Table 1. It was observed that the pregnant women showed similar maternal age, gestational age at birth, and pre-pregnancy weight (*p* > 0.05). The glucose, HbA1c, and placental index levels were higher in the GDM group (Table 1). Regarding the newborns, glycemic levels were similar, but the GDM group had a higher percentage of large-for-gestational-age newborns.

### 2.2. Placental Cytokines

**Figure 1:**
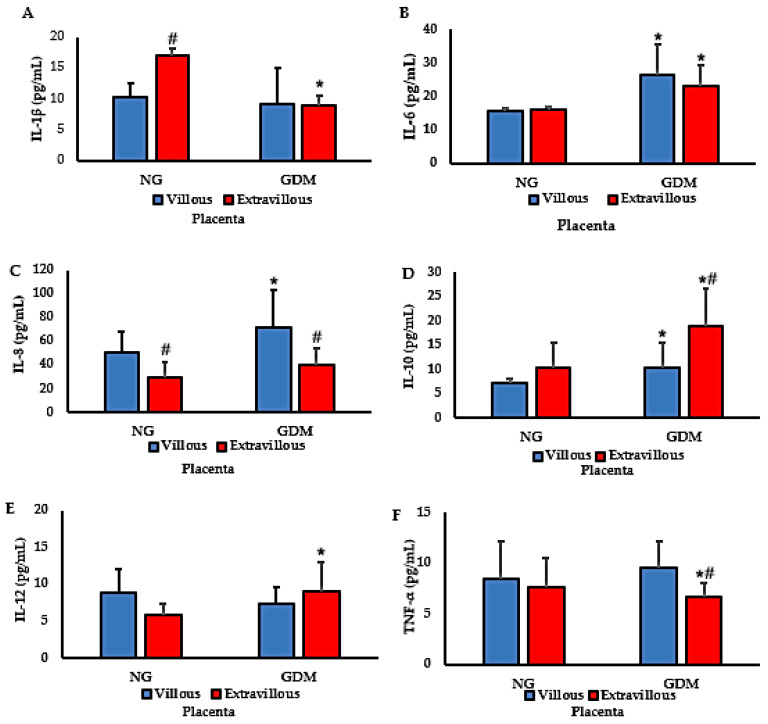
Cytokine concentrations in placentas from normoglycemic (NG) mothers and mothers with gestational diabetes mellitus (GDM). Panels: (**A**) IL-1β; (**B**) IL-6; (**C**) IL-8; (**D**) IL-10; (**E**) IL-12; (**F**) TNF-α. Data are expressed as mean ± standard error of the mean (SEM) from *n* = 20 placentas per group (NG and GDM), analyzed separately in villous (V) and extravillous (EV) compartments. Comparisons between groups (NG vs. GDM) and between placental regions (V vs. EV) were performed using independent-samples *t*-tests. **Symbols:** * *p* < 0.05 vs. NG of the corresponding region; # *p* < 0.05 villous vs. extravillous within the same group (NG or GDM).

Figure 1 presents cytokine concentrations in placentas from normoglycemic mothers and from mothers with gestational diabetes mellitus (GDM). IL-1β levels were reduced in the extravillous region of placentas from mothers with GDM. In contrast, placentas from normoglycemic mothers exhibited higher IL-1β concentrations in the villous region compared with the extravillous region, with the highest levels detected in the extravillous compartment (Figure 1A). IL-6 concentrations were increased in both villous and extravillous regions of placentas from mothers with GDM compared with normoglycemic controls (Figure 1B). Similarly, IL-8 levels were elevated in the villous region of placentas from mothers with GDM, where the highest concentrations were observed (Figure 1C).

For IL-10, increased levels were detected in placentas from mothers with GDM, with no differences between villous and extravillous regions within each group. The highest IL-10 concentrations were observed in the extravillous region of GDM placentas (Figure 1D). IL-12 levels were significantly higher in the extravillous region of placentas from mothers with GDM than in those from normoglycemic mothers. However, no differences were found between villous and extravillous regions within either group (Figure 1E). Finally, TNF-α levels were reduced in the extravillous region of placentas from mothers with GDM compared with normoglycemic mothers (Figure 1F).

**Figure 2:**
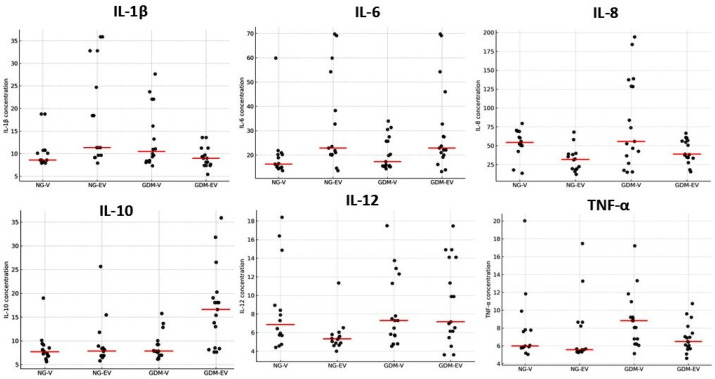
Dot plots showing individual cytokine concentrations (pg/mL) in villous (V) and extravillous (EV) placental regions from normoglycemic (NG) and gestational diabetes mellitus (GDM) mothers. Each dot represents one placenta. Data illustrate inter-individual dispersion for each cytokine (IL-1β, IL-6, IL-8, IL-10, IL-12, and TNF-α). The horizontal line (red) represents the median.

The dispersion of placental cytokine concentrations, as reflected in the vertical spread of points within each group, is shown in Figure 2. When comparing cytokine concentrations among the groups, a heterogeneous pattern of expression was observed across placental conditions and metabolic statuses. Overall, IL-1β exhibited low to moderate dispersion in the NG-EV and GDM-V groups, whereas IL-6 showed greater variability in the NG-EV and GDM-EV groups. IL-8 presented the highest degree of dispersion, particularly in the GDM-V group, while IL-10 displayed increased variability in the GDM-EV group. The cytokines IL-12 and TNF-α demonstrated moderate dispersion, with a slight increase in amplitude in both diabetic groups for IL-12 and, specifically, in the GDM-V group for TNF-α.

The placenta villous/extravillous cytokine ratio is shown in Table 2. The IL-1β and TNF-α ratios were higher, whereas the IL-8 ratio was lower in the GDM group. The ratios of IL-6, IL-10, and IL-12 were similar between the groups.

### 2.3. Immunophenotyping and Placental Macrophage Identification and Polarization

**Figure 3:**
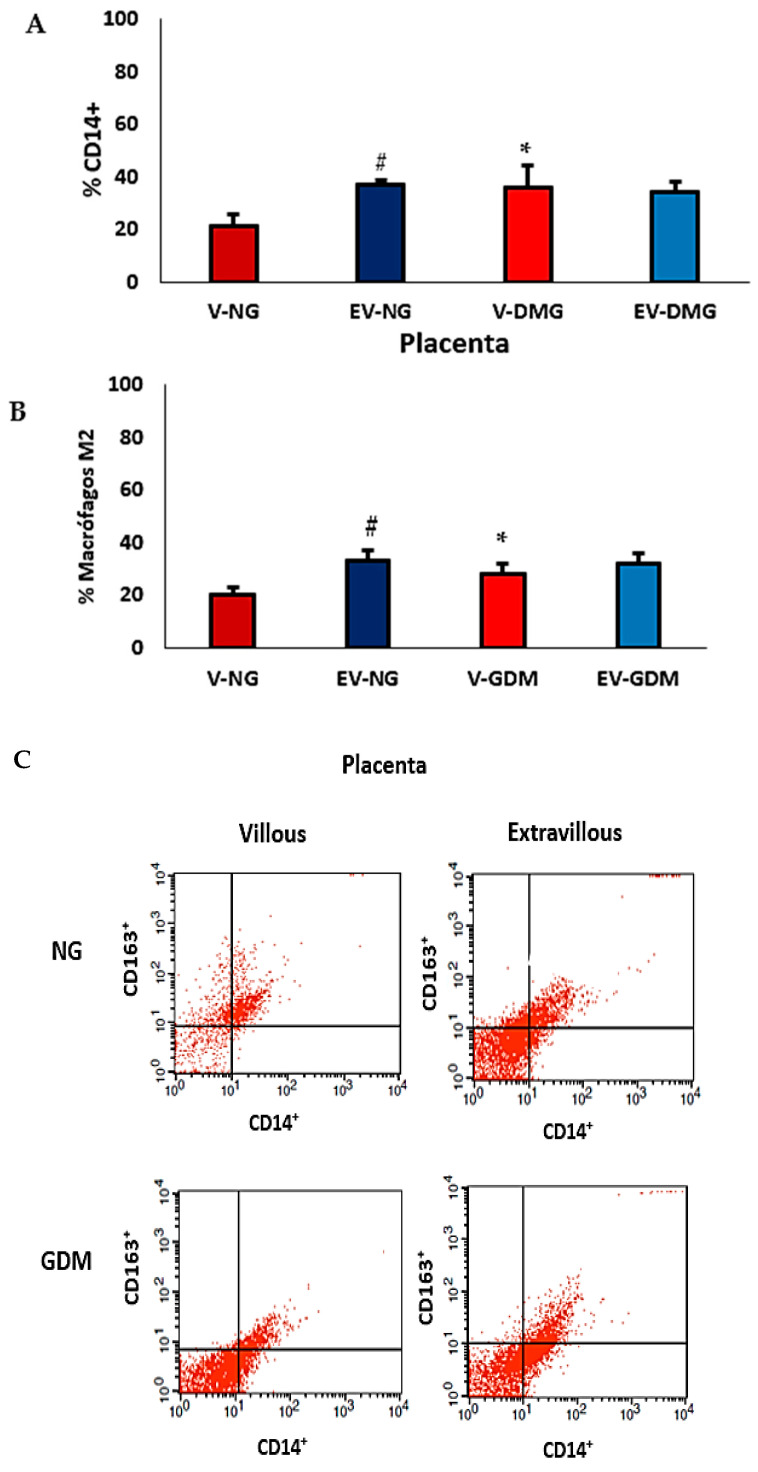
Percentage of CD14^+^ (**A**), CD14^+^CD163^+^ (**B**) macrophages, and (**C**) flow cytometric analysis of subsets of cells expressing CD14^+^CD163^+^ (M2 macrophages) in placentas from normoglycemic (NG) mothers and mothers with gestational diabetes mellitus (GDM). Villous (V) and extravillous (EV) regions were analyzed separately. Data are expressed as mean ± standard error of the mean (SEM) from placentas (n = 10) per group (NG and GDM). Comparisons between groups (NG vs. GDM) and between placental regions (V vs. EV) were performed using a two-tailed *t*-test for independent samples. Significance: *p* < 0.05. * indicates significant differences between NG and GDM groups within the same placental region; # *p* < 0.05 indicates significant differences between villous and extravillous regions within the same group.

Figure 3A shows an increased percentage of CD14^+^ cells in the villous region of placentas from mothers with GDM compared with the villous region of placentas from normoglycemic mothers (*p* < 0.05). Additionally, the proportion of CD14^+^ cells in the extravillous region of normoglycemic placentas was higher than that observed in the villous region (*p* < 0.05). No significant differences were found between villous and extravillous placental portions in the GDM group. Figure 3B shows an increased percentage of cells expressing CD14^+^CD163^+^ (M2) in the villous region of placentas from mothers with GDM. Also, the CD14^+^CD163^+^ cell population was higher in the extravillous placenta than in the villous region of normoglycemic mothers. Figure 3C shows flow cytometric analysis of CD14^+^CD163^+^ cell subsets.

**Figure 4:**
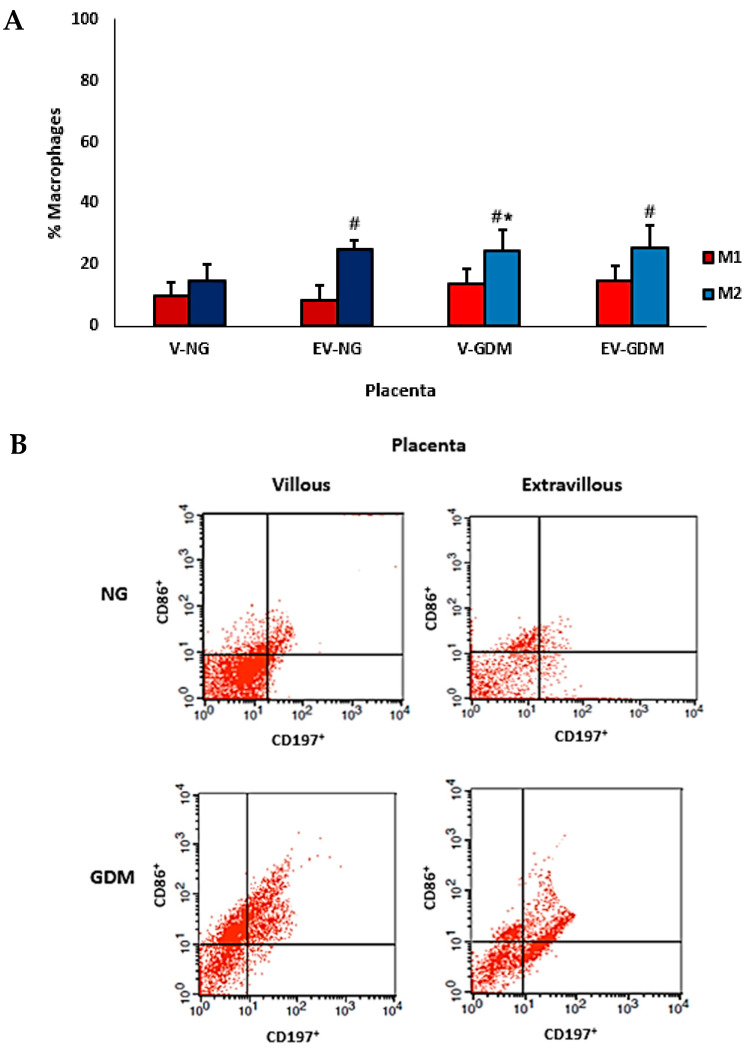
Percentage of M1 and M2 macrophage types (**A**) and flow cytometric analysis of subsets of cells expressing CD197^+^CD86^+^ (M1) and CD197^−^CD86^+^ (M2) macrophage types in placentas from normoglycemic (NG) mothers and mothers with gestational diabetes mellitus (GDM) (**B**). Villous (V) and extravillous (EV) regions were analyzed separately. Data are expressed as mean ± standard error of the mean (SEM) from n = 10 placentas per group (NG and GDM). Comparisons between groups (NG vs. GDM) and between placental regions (V vs. EV) were performed using independent-samples *t*-tests. * *p* < 0.05 indicates significant differences between the NG and GDM groups within the same placental region. # *p* < 0.05 indicates significant differences between villous and extravillous regions within the same group.

The comparison of macrophage subsets in placentas from mothers with GDM is shown in Figure 4A,B. An increase in M2 macrophage expression was observed in the villous region of placentas from mothers with GDM compared with the same region of placentas from normoglycemic mothers. When evaluating M1 and M2 macrophage percentages, an increase in M2 macrophage subtypes was observed in the villous and extravillous regions of placentas from mothers with GDM. Additionally, an increase in M2 macrophage expression was also detected in the extravillous region of placentas from normoglycemic mothers. In the villous region of placentas from normoglycemic mothers, the percentages of M1 and M2 macrophages were similar.

**Figure 5:**
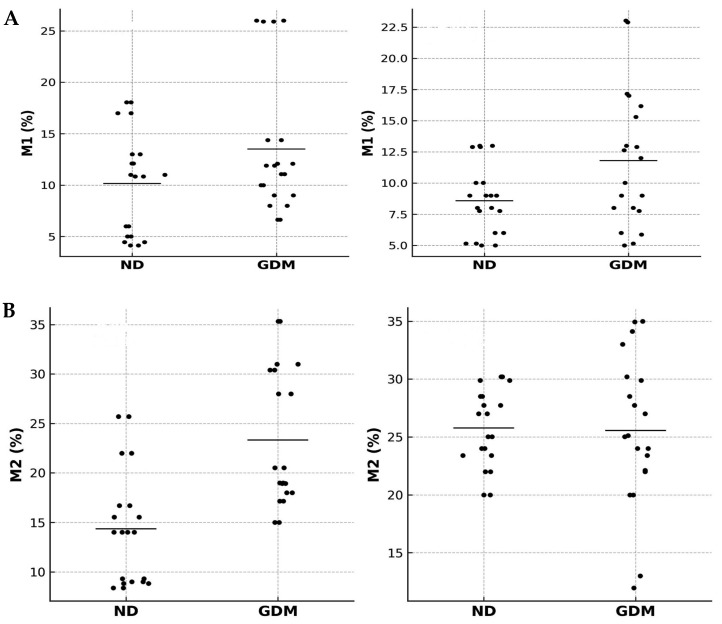
Correlation analyses of placental macrophages in normoglycemic (ND) and gestational diabetes mellitus (GDM) groups. Correlation analyses of M1 (**A**) and M2 (**B**) macrophages in villous and extravillous placental compartments from ND and GDM mothers. Scatterplots show Pearson correlation analyses of M1 or M2 macrophage percentages in villous and extravillous placental regions. Each dot represents an individual placenta (n = 20 per group). Black dots indicate ND and GDM samples, and horizontal black bars represent mean values for each group.

Figure 5, displaying the full distribution of the data points, Pearson’s *r* coefficients, and *p*-values for each comparison. The expression of macrophages M1 and M2 in the placenta showed distinct correlation patterns (Figure 5A). In the ND group, M1 macrophages displayed no significant correlation between villous and extravillous regions (r = 0.17; 95% CI −0.84 to 0.91; *p* = 0.786), whereas in the GDM group, a negative correlation was observed (r = −0.76; 95% CI −0.98 to 0.37; *p* = 0.014). When comparing groups, a significant negative correlation was detected in the villous compartment (r = −0.73; 95% CI −0.98 to 0.42; *p* = 0.016), while no correlation was found in the extravillous compartment (r = 0.23; 95% CI −0.82 to 0.92; *p* = 0.710).

Regarding M2 macrophages (Figure 5B), placentas from the ND group showed a negative correlation between villous and extravillous regions (r = −0.72; 95% CI −0.99 to 0.79; *p* = 0.028), whereas in the GDM group, the correlation was positive (r = 0.76; 95% CI −0.74 to 0.99; *p* = 0.024). Between groups, the villous compartment exhibited a negative correlation (r = −0.90; 95% CI, −1.00 to 0.47; *p* = 0.015), whereas the extravillous compartment showed a positive correlation (r = 0.67; 95% CI, −0.82 to 0.99; *p* = 0.033).

In the integrated analysis (M1 vs. M2), no significant correlations were detected. In the villous compartment, both ND (r = −0.15; 95% CI −0.91 to 0.84; *p* = 0.809) and GDM (r = −0.58; 95% CI −0.97 to 0.62; *p* = 0.301) groups showed no association. Similarly, in the extravillous compartment, correlations were absent in both ND (r = −0.41, 95% CI −0.95 to 0.74, *p* = 0.496) and GDM (r = −0.48, 95% CI −0.96 to 0.70, *p* = 0.411).

**Figure 6:**
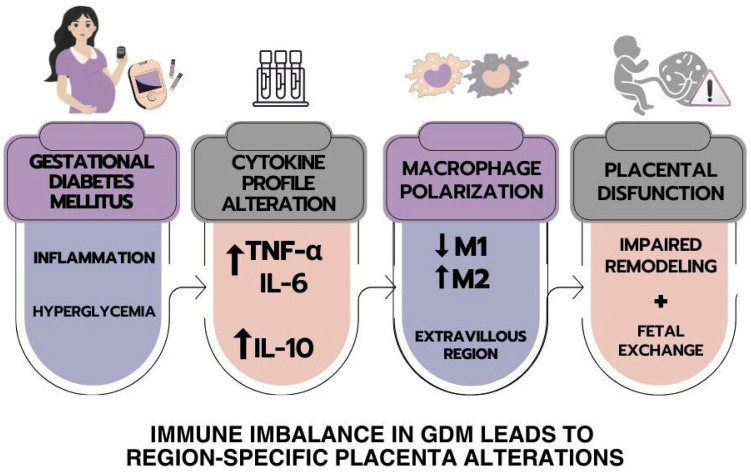
The schematic representation summarizes the main findings in the human placenta from mothers with GDM. The cytokines, M1 and M2 macrophages, and their correlations in the placentas of women with gestational diabetes mellitus are presented.

The integrative representation shown in Figure 6 summarizes the main findings illustrating the immunological cascade triggered by gestational diabetes mellitus (GDM). In this schematic, GDM is depicted as a state of metabolic and inflammatory stress, leading to altered cytokine profiles characterized by elevated TNF-α, IL-6, and IL-10. These alterations are linked to macrophage polarization dynamics in the extravillous region, with a relative decrease in M1-like and predominance of M2-like macrophages.

## 3. Discussion

Diabetes is a major public health concern, particularly in developing countries, where the highest prevalence and incidence rates are reported [27]. Its impact arises not only from hyperglycemia-related clinical manifestations but also from functional alterations across multiple organs and systems resulting from chronic dysregulation of protein and lipid metabolism. In this study, we evaluated cytokine levels and macrophage polarization in placentas from mothers with gestational diabetes mellitus (GDM). Our findings indicate that maternal hyperglycemia modulates cytokine expression and the balance between M1 and M2 macrophages, with distinct patterns observed in villous and extravillous placental compartments.

The inflammatory response triggered by hyperglycemia, together with maternal immune activity, may represent a key mechanism leading to neonatal complications. Altered cytokine levels have been consistently reported in both blood and placental tissue from diabetic mothers [4,5]. In our study, maternal hyperglycemia was associated with distinct cytokine profiles across placental regions. The reduction of IL-1β and TNF-α in the extravillous compartment of GDM placentas is particularly relevant, since these cytokines regulate maternal–fetal immune interactions, vascular remodeling, and trophoblast invasion [28,29].

Studies on GDM placentas have reported conflicting cytokine patterns. Some described increased IL-1β and TNF-α expression in both maternal and fetal compartments [18,30], whereas others, consistent with our data, observed downregulation of these cytokines at the decidual interface [14]. Such discrepancies may reflect differences in sampling methods, gestational age, or glycemic control.

From a functional perspective, reducing IL-1β and TNF-α in the extravillous region may attenuate local inflammation and protect trophoblasts from excessive immune activation; however, this downregulation may come at the cost of incomplete spiral artery remodeling and suboptimal placental perfusion. This duality highlights the placenta’s effort to balance immune tolerance and vascular remodeling under hyperglycemic stress [18,31]. Thus, our data demonstrate not only cytokine imbalance but also its functional implications for placental homeostasis.

Cytokines such as IL-1β, TNF-α, and IL-6 play essential roles in trophoblast proliferation, vascular adaptation, and maternal–fetal communication. In GDM, their regional dysregulation suggests localized remodeling of the placental microenvironment rather than a uniform inflammatory state. Although GDM is associated with systemic elevations in IL-6 [31], local downregulation of these cytokines at the placental interface may represent a compensatory mechanism to prevent excessive inflammation and protect fetal tissues [32]. Nevertheless, persistent inflammation can impair spiral artery remodeling, compromise uteroplacental circulation, and disrupt oxygen and nutrient delivery [33].

Interestingly, IL-1β levels were higher in the villous region of normoglycemic placentas, emphasizing the physiological role of this cytokine in trophoblast–endothelial interactions during pregnancy [4,34]. TNF-α, while essential in early gestation, is also implicated in insulin resistance and metabolic dysregulation in GDM [35]. This dual role may explain why excessive TNF-α contributes to preterm labor, whereas balanced levels support normal gestation [36].

We also observed increased IL-6 in both villous and extravillous compartments, upregulation of IL-8 in the villous portion, and elevated IL-12 in the extravillous region of GDM placentas. IL-6, a pleiotropic cytokine associated with chronic inflammation and insulin resistance [29,31], has been linked to adverse perinatal outcomes [37,38]. Upregulation of IL-8 in the villi suggests enhanced neutrophil recruitment and angiogenic signaling, consistent with villous abnormalities associated with metabolic disorders [39]. Elevated IL-12 in the extravillous region indicates a shift toward Th1-type immunity, potentially impairing maternal–fetal tolerance and contributing to preeclampsia and fetal growth restriction [40,41]. Conversely, increased IL-10 in the extravillous compartment of GDM placentas may represent a compensatory mechanism to preserve placental function [31,42,43].

Taken together, these findings suggest that GDM involves a complex immunoinflammatory component characterized by downregulation of IL-1β and TNF-α in the extravillous compartment and upregulation of IL-6, IL-8, IL-12, and IL-10 in specific placental regions. The villous-to-extravillous cytokine ratio further distinguished GDM placentas, showing higher IL-1β and TNF-α ratios and lower IL-8 ratios in the villous portion. This selective modulation reflects the placenta’s attempt to balance inflammation and tolerance but may impair vascular remodeling and nutrient exchange.

In addition to cytokine alterations, immune cell subsets are also affected in diabetes [6,26,43,44,45,46]. Macrophages are central to these changes because they can polarize into proinflammatory (M1) or anti-inflammatory (M2) phenotypes [47]. In human placental macrophages, CD163 is recognized as a distinctive surface marker [47], and an increased number of CD163^+^ cells has been observed in villous and intervillous compartments of placentas from pregnancies with fetal growth restriction [8]. Furthermore, studies have identified CD14^+^CD163^+^ Hofbauer cells as a key M2-type population within the human placenta [48].

In this study, the increased proportion of CD14^+^ cells in the villous region of GDM placentas suggests enhanced recruitment or persistence of monocyte/macrophage lineage cells in response to hyperglycemia. This may reflect a compensatory mechanism that preserves tissue remodeling and immune homeostasis under metabolic stress. Conversely, the higher CD14^+^ cell frequency in the extravillous region of normoglycemic placentas indicates the physiological predominance of macrophages in areas of trophoblast invasion and vascular remodeling.

The elevated proportion of CD14^+^CD163^+^ (M2) macrophages in the villous region of GDM placentas points to a local shift toward an anti-inflammatory or tissue-repair profile. While this may protect against excessive inflammation and oxidative stress, an excessive M2 bias could impair the proinflammatory signaling necessary for adequate vascular adaptation. Overall, these findings indicate that GDM disrupts the regional balance of macrophage polarization, leading to altered immune organization within the placenta and potentially contributing to impaired maternal–fetal exchange and suboptimal fetal outcomes [31,47,49].

The predominance of proinflammatory, classically activated (M1-type) macrophages in placentas from mothers with GDM has been linked to shallow trophoblast invasion and impaired vascular remodeling [8,12]. In contrast, our findings revealed an enrichment of CD14^+^CD163^+^ M2-type macrophages in the villous compartment of GDM placentas. It is important to note that most previous studies evaluated the placenta as a whole or focused primarily on decidual or anchoring regions. In contrast, our compartment-specific analysis (villous vs. extravillous) allows a more precise assessment of local microenvironmental adaptations.

Differences in clinical context may also account for discrepancies among studies. Our samples were collected at term from mothers with established GDM but without preeclampsia, whereas studies reporting M1 predominance often involved conditions of greater metabolic or inflammatory stress and hypertensive comorbidities. In this context, the predominance of M2 macrophages in the villous region observed in our study likely represents a compensatory response to chronic hyperglycemia and cytokine imbalance. This interpretation is further supported by the concomitant increases in IL-6 and IL-8 within the villous compartment, as well as by previous reports describing CD163^+^ macrophages as anti-inflammatory and tissue-remodeling phenotypes in the human placenta.

Although M2 macrophages are classically associated with tissue repair and immune tolerance [50,51], their redistribution toward the villous region in GDM may alter normal placental physiology, potentially affecting trophoblast–endothelial interactions and nutrient exchange. The parallel rise in IL-6 and IL-8 in these regions reinforces this notion [52]. Interestingly, when comparing macrophage subsets, we observed a significant increase in M2 cells in both villous and extravillous compartments of GDM placentas, as well as in the extravillous region of normoglycemic placentas. This pattern underscores the remarkable plasticity of the placental immune environment, which adapts to maternal immunometabolic conditions by dynamically modulating macrophage activation in response to both physiological and pathological stimuli.

Correlation analysis further supports this interpretation. The results indicate an imbalance between M1 and M2 profiles in GDM, characterized by a predominance of negative correlations. Specifically, M2 macrophages displayed opposite correlation patterns between groups, showing negative correlations in normoglycemic placentas and positive correlations in GDM. In contrast, M1 macrophages exhibited more consistent negative correlations in GDM, particularly within the villous regions. The analysis of M1–M2 relationships also suggested a functional imbalance in GDM. These findings suggest that hyperglycemia promotes divergent regulatory behavior of macrophages across placental compartments, reflecting their functional plasticity and context-dependent responses.

Nevertheless, excessive M2 polarization in GDM may not always be protective. Under pathological conditions, M2 macrophages may acquire hybrid phenotypes, retaining some proinflammatory activity while maintaining anti-inflammatory traits, which may contribute to placental dysfunction [49,53]. Thus, the M2 predominance observed in GDM may represent both a compensatory and maladaptive response.

Our findings demonstrate region-specific alterations in macrophage polarization and cytokine balance in GDM, revealing localized immune remodeling within the placenta. This compartmental disruption is biologically relevant, as placental macrophages regulate trophoblast invasion, spiral artery remodeling, and uteroplacental perfusion. An imbalance toward either a proinflammatory or an alternatively activated profile may therefore contribute to defective vascular adaptation—a key mechanism shared by placental insufficiency and preeclampsia [12,54,55,56,57].

In GDM, hyperglycemia-induced inflammation alters macrophage activation and cytokine production, potentially linking metabolic stress to abnormal placental structure and function. The coexistence of villous inflammation with elevated IL-6 and IL-8, along with M2 predominance, suggests an adaptive—but potentially maladaptive—attempt to maintain tissue repair and nutrient exchange. While M2 polarization may initially preserve placental integrity, excessive tolerance or repair signaling could promote villous overgrowth and inefficient perfusion. Conversely, reduced IL-1β in the extravillous compartment may limit cytotoxic inflammation but also hinder proper vascular remodeling.

Together, these findings support a model in which hyperglycemia induces cytokine-driven macrophage reprogramming, resulting in region-specific immune imbalance and vascular dysfunction. This immune–metabolic crosstalk provides a mechanistic basis linking GDM to abnormal fetal growth, impaired perfusion, and increased risk of hypertensive disorders.

Despite some variability reflecting intrinsic biological heterogeneity, our results highlight placental macrophage polarization as a potential mediator of the relationship between maternal metabolic state and local immune adaptation. Future studies should clarify whether macrophages from GDM placentas directly affect trophoblast invasion, endothelial integrity, and nutrient transport, confirming their causal role in the pathophysiology of GDM-related placental dysfunction.

Additionally, our analysis was restricted to term placentas, which may limit extrapolation of results to earlier stages of pregnancy. Immunophenotyping and cytokine quantification provide valuable insights into immunological profiles; however, further studies exploring dynamic interactions and cytokine kinetics in vivo are warranted. Moreover, the cross-sectional design prevents establishing causality between GDM and macrophage polarization, an important consideration given the multifactorial nature of pregnancy complications and the contribution of systemic maternal inflammation [58].

Overall, our findings are consistent with previous studies demonstrating region-specific alterations in placental macrophage polarization in GDM [4,5,44,45,59,60,61,62]. The predominance of M2 phenotypes may initially act as a compensatory mechanism against hyperglycemia-induced inflammation, but excessive or functionally altered M2 cells may impair placental exchange and fetal development. A deeper understanding of these mechanisms may support the development of targeted interventions to improve maternal–fetal outcomes in diabetic pregnancies.

## 4. Materials and Methods

### 4.1. Study and Subjects

Placentas from mothers with gestational diabetes mellitus were evaluated in a cross-sectional study. The subjects attended the Diabetes and Pregnancy Facility, the School of Medicine Obstetrics Course at Unesp, Botucatu, SP. Placental samples from pregnant women aged 18–45 years were analyzed according to maternal glycemic status. All participants underwent a 75-g oral glucose tolerance test (OGTT-75g) [27] and a glucose profile (GP) [63], performed in parallel between the 24th and 28th weeks of gestation. An altered GP was defined as fasting glycemia ≥ 90 mg/dL or postprandial glycemia ≥ 130 mg/dL [64]. The OGTT-75g was considered abnormal when any of the following plasma glucose thresholds were met or exceeded: fasting ≥ 92 mg/dL, 1 h postload ≥ 180 mg/dL, or 2 h postload ≥ 153 mg/dL [63].

Based on OGTT-75g and GP results, 40 pregnant women were selected and classified into two groups: non-diabetic (ND; normal OGTT-75g and GP, n = 20) and gestational diabetes mellitus (GDM; abnormal OGTT-75g, n = 20) [64]. All women continued routine follow-up at the facility, regardless of diagnosis, and patients with hyperglycemia received specific glycemic control management [64].

### 4.2. Subject Follow-Up and Characterization

Glycemic control was monitored throughout pregnancy. Glycemic control data were available for all GDM participants, who followed standard obstetric management protocols including dietary counseling and, when necessary, insulin therapy. All cases analyzed achieved adequate glycemic control by clinical criteria. Adequate control was defined as a mean glycemia ≤ 120 mg/dL, while inadequate control corresponded to a mean glycemia > 120 mg/dL. Women with GDM received individualized dietary recommendations and exercise counseling, and insulin therapy was initiated when glycemic control was inadequate [4]. ND the pregnant women underwent any intervention for glycemic control.

### 4.3. Placenta Sampling and Preparation of Macrophages

The placenta was collected at the moment of delivery and immediately washed with a saline solution. Samples of villous tissue were collected from areas midway between the maternal and fetal sides of the placenta, avoiding infarcted or calcified regions. Large vessels were carefully removed, and only terminal villi were retained [4,64]. The basal plate was then dissected under a stereomicroscope to separate the extravillous trophoblastic layer from the underlying villous tissue and the amniotic membrane, ensuring minimal cross-contamination. Isolation of extravillous tissue followed an adaptation of the previously described protocol for amnio-chorion cytotrophoblast separation [64,65].

To ensure compartmental purity, tissue fragments were visually inspected, and purity was confirmed by microscopic evaluation of hematoxylin–eosin-stained sections, identifying characteristic features of villous (chorionic villi) and extravillous (decidual and anchoring trophoblast) structures. Isolated cells were assessed for viability using the trypan blue exclusion method (>95% viability was required for subsequent flow cytometry).

Placental fragments were immediately stored in liquid nitrogen and later processed for cell separation. Placental fragments were macerated in PBS with 0.05% Tween-20 supplemented with protease inhibitors (0.1 mM of phenylmethylsulfonyl fluoride; 0.1 mM of benzethonium chloride, 10 mM of EDTA, 20 UI of aprotinin, and 0.5% of BSA) in a proportion of 100 mg of tissue/mL, using a homogenizer Power Gen 125 (Fisher Scientific@) (Waltham, MA, USA). The homogenate was filtered and reserved for cytokine quantification, while the sediment (cells) was fractionated by centrifugation (160× *g*, 40 min) through a density gradient. The isolation procedure was performed by Ficoll-Paque (density 1.077 g/L—Sigma Chemical, St. Louis, MO, USA). After centrifugation, the supernatant was discarded, and the opaque bands at the interfaces between PBS and Ficoll-Paque-1077 containing cells were collected and transferred to tubes. Mononuclear cells were washed twice with 199 medium culture (Sigma Chemical, St. Louis, MO, USA). The cells were used immediately for immunophenotyping assays.

### 4.4. Quantification of Cytokines

Placenta homogenate was collected and stored at −80 °C for subsequent analysis. Cytokine concentrations were measured using the BD™ Cytometric Bead Array (CBA) Human Inflammatory Cytokine Kit (BD Biosciences, San Jose, CA, USA; Cat. No. 551811), following the manufacturer’s instructions. The panel simultaneously quantifies IL-1β, IL-6, IL-8, IL-10, IL-12p70, and TNF-α. Samples were analyzed using flow cytometry (FACS Calibur, BD Bioscience, USA). The results were generated using BD CellQuest software, version 5.1, and the data were analyzed using BD FCAP Array™ software (version 3.0). The lower limits of detection for each analyte were: IL-1β = 2.3 pg/mL; IL-6 = 1.6 pg/mL; IL-8 = 1.2 pg/mL; IL-10 = 0.13 pg/mL; IL-12p70 = 0.6 pg/mL; TNF-α = 0.7 pg/mL. Samples below the detection threshold were assigned the lowest detectable value for statistical analysis. All samples were processed and analyzed under identical conditions to minimize inter-assay variability.

Placental cytokine concentrations were normalized to the wet weight of tissue processed to account for variability among samples. Each homogenate corresponded to 100 mg of placental tissue per 1 mL of buffer, ensuring equivalent dilution across all samples. To verify homogenization consistency, total protein was determined by the Biuret colorimetric method [66]. Cytokine concentrations were expressed as pg/mL per 100 mg of tissue, allowing for direct comparison between placentas and regions.

### 4.5. Immunophenotyping Macrophage Identification and Polarization

Placenta cells were washed with Phosphate Buffer (PBS) plus BSA (bovine serum albumin) for 10 min at 4 °C. Cells were labeled with 5 μL of anti-CD14^+^ (FITC). A PE-tagged IgG1 isotype was used as a control. Cells were evaluated by flow cytometry. Cells expressing CD14^+^ were used for polarization analysis [67,68]. Cell suspensions were labeled with antibodies specific for CD197, CD86, and CD163, and then fixed and permeabilized with Cytofix-Cytoperm solution (BD Biosciences, USA).

Macrophage subsets were identified from placental cell suspensions using surface markers for M1 (CD197^+^/CD86^+^) and M2 (CD197-CD86^+^ or CD14^+^/CD163^+^) phenotypes. The gating strategy followed a sequential approach: first, debris and doublets were excluded using FSC/SSC parameters, followed by selection of live CD14^+^ monocytes/macrophages [46,47].

### 4.6. Statistical Analysis

Data are presented as mean ± standard deviation. A *priori* power analysis was performed to estimate the minimum required sample size. Assuming a moderate effect size (Cohen’s *d* ≈ 0.8) for differences in cytokine expression and macrophage polarization between normoglycemic and GDM placentas, with α = 0.05 and a statistical power of 0.80 for a two-tailed test, a minimum of 10 samples per group was determined.

The D’Agostino test was used to assess data normality. Comparisons between two independent groups were conducted using Student’s *t*-test. Differences in cytokine concentrations and the percentages of M1 and M2 macrophages in villous and extravillous placental regions were analyzed. Correlations between M1 and M2 macrophages were evaluated using Pearson’s linear correlation. Statistical significance was set at *p* < 0.05.

## 5. Conclusions

This study reinforces that gestational diabetes mellitus (GDM) induces significant immune alterations in the placenta, particularly through region-specific changes in macrophage polarization and cytokine production. The villous compartment exhibited a predominant proinflammatory profile, characterized by elevated M1 markers and increased levels of TNF-α and IL-6. In contrast, the extravillous region demonstrated a more heterogeneous pattern with partial preservation of M2 markers. These findings support the concept that placental immune responses are spatially regulated, underscoring the relevance of localized immune imbalance in the pathogenesis of GDM. The identified alterations may contribute to impaired placental function and adverse pregnancy outcomes. Understanding these compartment-specific immune dynamics provides important perspectives for the development of targeted interventions at the maternal-fetal interface in diabetic pregnancies.

## Figures and Tables

**Table 1:** Maternal and neonatal clinical data from the non-diabetic (ND) and gestational diabetes mellitus (GDM) groups.

Parameters	ND	GDM	*p*-Value
Maternal			
Age (years)	26.4 ± 4.1	28.6 ± 6.7	0.4421
Gestational age (weeks)	39.2 ± 0.5	38.3 ± 0.9	0.0882
Mean glycemia (mg/dL)	81.9 ± 6.7	100.1 ± 11.6 *	0.0341
HbA1c (%)	4.9 ± 0.5	5.8 ± 0.7 *	0.0287
BMI (1st trimester)	26.9 ± 6.2	28.5 ± 7.8	0.7846
BMI (3rd trimester)	31.1 ± 7.7	32.4 ± 6.4	0.8237
Newborn			
Glucose	77.6 ± 7.9	66.5 ± 14.1	0.1778
Birth weight (%)			
SGA	15% (3)	5% (1)	
AGA	75% (15)	70% (14)	
LGA	10% (2)	25% (5)	
Placental weight (g)	609.3 ± 87.4	706.7 ± 92.7	0.7025
Placental weight/fetal weight ratio	0.167 ± 0.032	0.180 ± 0.037 *	0.0032

**Notes:** NG—normoglycemic; GDM—gestational diabetes mellitus. HbA1c—glycated hemoglobin; BMI (1st trimester)—body mass index in the first trimester of pregnancy; BMI (3rd trimester)—body mass index in the third trimester of pregnancy; SGA—small for gestational age; AGA—adequate for gestational age; LGA—large for gestational age. Data are expressed as mean ± standard deviation (SD). * Indicates significant differences between groups.

**Table 2:** Placental villous/extravillous cytokine ratio in groups without diabetes (ND) and gestational diabetes mellitus (GDM).

Placental Cytokines	Villous Layer/Extravillous Layer Ratio		*p*-Value
	ND	GDM	
IL-1 β	0.73 ± 0.2	1.08 ± 0.21 *	0.0402
IL-6	0.77 ± 0.25	0.79 ± 0.23	0.2663
IL-8	2.0 ± 0.27	1.41 ± 0.81 *	0.0473
IL-10	0.87 ± 0.24	0.71 ± 0.33	0.1237
IL-12	1.45 ± 0.61	1.08 ± 0.59	0.1283
TNF-α	0.97 ± 0.17	1.55 ± 0.61 *	0.0325

Note: Results are expressed as the mean of samples of placental tissue. * Statistical difference between groups.

## Data Availability

The data supporting the findings of this study are available from the corresponding authors upon request.
